# tRNA, yRNA, and rRNA fragment excisions do not involve canonical microRNA biogenesis machinery

**DOI:** 10.17912/micropub.biology.001332

**Published:** 2024-11-19

**Authors:** Noel L Godang, Anita D Nguyen, Jeffrey D DeMeis, Sunita S Paudel, Nick J Campbell, Kingston J Barnes, Kahyeon Jeon, Alayla S Roussell, Kimberly A Gregson, Glen M Borchert

**Affiliations:** 1 Pharmacology, University of South Alabama College of Medicine, Mobile, AL; 2 Computer Science, University of South Alabama School of Computing, Mobile, AL; 3 Alabama School of Mathematics and Science

## Abstract

The excision of specific tRNA-derived small RNAs (tsRNAs), yRNA-derived small RNAs (ysRNAs) and ribosomal RNA-derived small RNAs (rsRNAs) is now well established. Several reports have suggested many of these fragments function much like traditional microRNAs (miRNAs). That said, whereas the expressions of the majority of appreciably expressed miRNAs in HCT116 colon cancer cells are significantly decreased in individual knockouts (KOs) of DROSHA, DGCR8, XPO5, and DICER, on average, only 3.5% of tsRNA, ysRNA, and rsRNA expressions are impaired. Conversely, tsRNA, ysRNA, and rsRNA expressions are significantly increased in each of these KOs as compared to WT. As such, although DICER has been suggested to be involved with the expression of specific tsRNAs, ysRNAs, and rsRNAs, our study finds no evidence supporting the involvement of any of these canonical miRNA biogenesis enzymes in their expressions.

**Figure 1. MiRNA, tsRNA, rsRNA, and ysRNA expressions in canonical miRNA processing enzyme knockouts (KOs):**
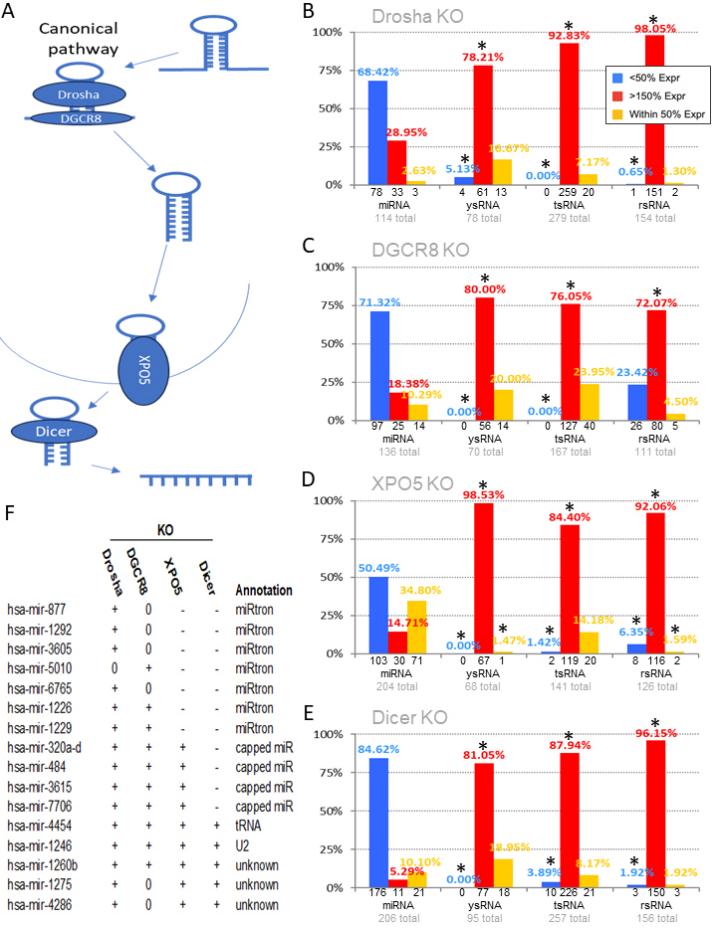
( **A** ) Cartoon of canonical miRNA processing. Adapted from (King and Borchert 2017). ( **B-E** ) Human colon cancer cell line (HCT116) WT and KO small RNA transcriptome comparisons. ( **B** ) Graph depicting the percentage of (from left to right) miRNA, ysRNA, tsRNA and rsRNA expressions that are significantly decreased (blue), increased (red), or unchanged (orange) in DROSHA-KO cells in comparison to WT. *, p < 0.0001 as compared to miRNAs. ( **C** ) Graph as in B in DGCR8-KO. ( **D** ) Graph as in B in XPO5-KO. ( **E** ) Graph as in B in DICER-KO. ( **F** ) Select miRNAs with expressions increased in at least one indicated KO. +, expression increased by ≥50% of WT. 0, expression within 50% of WT. -, expression decreased by ≥50% of WT. Annotations as reported in: Mirtron (Da Fonseca, Domingues, and Paschoal 2019), capped miRNA (Kim, Kim, and Kim 2016), tRNA (Reinsborough et al. 2019), and U2 (Xu et al. 2019).

## Description

It is now clear that over 75% of our genes encode RNA transcripts that never get translated to protein. The cellular pool of these noncoding RNAs (ncRNAs) is highly complex in terms of its diversity and function. Different structural and functional classes of ncRNAs act as regulators of key cellular processes, many of which are associated with disease (Danielson et al. 2017; Müller et al. 2015; MV et al. 2021) . Due to early identification of essential regulatory roles for microRNAs (miRNAs) in cell proliferation, differentiation, apoptosis, and metabolism, miRNAs have constituted one of the most actively researched classes of ncRNAs for much of the last 25 years, and as such, it is now well established that alterations in miRNA function are frequently involved in the pathogenesis of many human diseases (John et al. 2004; Ma 2016; Chevalier and Borchert 2017; Giglio et al. 2021) .

MiRNAs are transcribed by RNA Polymerase II or III (Graves and Zeng 2012) as an initial transcript (deemed a pri-miRNA) often several thousands of base pairs in length containing an internal hairpin (Li et al. 2009) . The pri-miRNA is next processed by the Microprocessor ribonuclease protein complex composed of DROSHA (which targets and cleaves the flanking ends of the hairpin) and DGCR8 (which stabilizes the complex on the pri-miRNA). DROSHA processing yields an ~100 nucleotide (nt) long stem loop called a precursor miRNA or pre-miRNA (Yue et al. 2011) . Following excision, the pre-miRNA stem loop is transported out of the nucleus and into the cytoplasm via the transport protein exportin-5 (XPO5). Once in the cytoplasm, the pre-miRNA is targeted by another ribonuclease, DICER, which cleaves the molecule further by removing the loop portion of the hairpin leaving an intermediate duplex (~20 base pairs in length). Finally, the passenger strand of this duplex is discarded and the final mature miRNA is loaded into an Argonaute (Ago) protein (Graves and Zeng 2012) ( **Figure 1A** ).

That said, recent transcriptomic studies, made possible by the development of new Next Generation Sequencing (NGS) technologies, have identified large numbers of miRNA-sized fragments excised from longer ncRNAs that possess their own well characterized cellular activities (Macias et al. 2012; Cole et al. 2009; Kasukurthi et al. 2019) . Named according to the RNA from which they are excised, tRNA-derived small RNAs (tsRNAs) represent perhaps the best studied small ncRNA fragments (sncRNAs) to date, and accumulating evidence suggests that these RNA fragments are themselves physiologically relevant in health and disease (Alexander Bishop Coley et al. 2022; Alexander B. Coley et al. 2022; Cao, Cowan, and Wang 2020). TSRNAs were first recognized as a class of functional sncRNAs in 2009 (Lee et al. 2009; Wang et al. 2013; Martens-Uzunova, Olvedy, and Jenster 2013) and have now been identified in all domains of life (Krishna et al. 2021) . Despite this, although some tsRNAs have been reported to silence mRNAs through an RNAi mechanism much like miRNAs (Zhou et al. 2017; Venkatesh, Suresh, and Tsutsumi 2016) , how tsRNAs are generated and the functional roles of the majority of tsRNAs remain unclear. Similarly, another class of sncRNAs whose fundamental biogenesis remains largely undefined, yRNA fragments termed yRNA-derived small RNAs (ysRNAs) have now also been reported to function in stress, apoptosis and cancer much like miRNAs (Billmeier et al. 2022) . Further, the most highly expressed cellular RNAs, ribosomal RNAs (rRNAs), are also processed to generate specific rRNA-derived small RNAs (rsRNAs) which can immunoprecipitate with Ago complexes in human and mouse cells (Guan and Grigoriev 2021) . However, despite the mounting body of evidence indicating that sncRNA fragments derived from longer ncRNAs are themselves physiologically relevant, little is known about how specific sncRNAs are selected for excision from longer ncRNAs nor about how these ncRNA fragments are directed to engage in post-transcriptional regulations distinct from the activities of their longer ncRNA progenitors.

In light of numerous reports suggesting that (1) many tsRNAs, ysRNAs, and rsRNAs function much like mature miRNAs, and (2) that DICER might be involved with their biogenesis (recently reviewed in (Akiyama and Ivanov 2023) ), we elected to examine tsRNA, ysRNA, and rsRNA expressions in knockouts (KOs) of canonical miRNA processing enzymes ( **Figure 1A** ). Independent analysis of the small RNA transcriptomes of human colon cancer HCT116 WT and DROSHA-KO cells identified 114 miRNAs, 78 ysRNAs, 154 rsRNAs, and 279 tsRNAs to be appreciably expressed (≥ 20 transcripts per million (TPM)) in WT and/or KOs. Notably, whereas we find the expressions of 78 of 114 miRNAs to be significantly decreased in HCT116 DROSHA-KOs, we find 61 of 78 ysRNA, 151 of 154 rsRNA, and 259 of 279 tsRNA expressions to be conversely, significantly increased in DROSHA-KO HCT116 cells as compared to WT ( **Figure 1B** ). Similarly, analysis of the HCT116 DGCR-KO small RNA transcriptome identified 136 miRNAs, 70 ysRNAs, 111 rsRNAs, and 167 tsRNAs appreciably expressed. Whereas we find the expressions of 97 of 136 miRNAs to be significantly decreased in HCT116 DGCR8-KOs, we find 56 of 70 ysRNA, 80 of 111 rsRNA, and 127 of 167 tsRNA expressions to be conversely, significantly increased in DGCR8-KO HCT116 cells as compared to WT ( **Figure 1C** ). Strikingly, we found no tsRNA expressions were increased in either DROSHA or DGCR8 KOs ( **Figure 1B,C** ). Further, analysis of the HCT116 XPO5-KO small RNA transcriptome identified 204 miRNAs, 68 ysRNAs, 126 rsRNAs, and 141 tsRNAs appreciably expressed. Notably, whereas we find the expressions of 103 of 204 miRNAs to be significantly decreased in HCT116 XPO5-KOs, we find 67 of 68 ysRNA, 116 of 126 rsRNA, and 119 of 141 tsRNA expressions to be conversely, significantly increased in XPO5-KO HCT116 cells as compared to WT ( **Figure 1D** ). Finally, analysis of the HCT116 DICER-KO small RNA transcriptome identified 208 miRNAs, 95 ysRNAs, 156 rsRNAs, and 257 tsRNAs appreciably expressed. Whereas we find the expressions of 176 of 208 miRNAs to be significantly decreased in HCT116 DICER-KOs, we find 77 of 95 ysRNA, 150 of 156 rsRNA, and 226 of 257 tsRNA expressions to be conversely, significantly increased in DICER-KO HCT116 cells as compared to WT ( **Figure 1E** ). It should be noted that while they were not appreciably decreased like miRNAs, the apparent upregulations of many tsRNAs, ysRNAs, and rsRNAs in the miRNA-biogenesis related KOs is in large part due to miRNA depletion leading to other small RNAs assuming larger percentages of the reads (Shi, Zhou, and Chen 2022) .

Importantly, our results strongly agree with previous analyses involving some of these same small RNA transcriptomes. In 2016, Kim et al. (Kim, Kim, and Kim 2016) similarly identified hsa-miRs -320(a, b1, b2, c1, d1, d2), -484, -3615, and -7706 as capped miRNAs only requiring canonical DICER (and not DROSHA or XPO5) for expression. In contrast, they also found hsa-miR-877 (a known mirtron) required DICER and XPO5 (but not DROSHA) for expression. In addition to hsa-miR-877, we find similar requirements for one or both members of the microprocessor complex for more recently described human mirtron miRNAs (hsa-miRs -1226, -1229, -1292, -3605, -5010, and -6765) (Da Fonseca, Domingues, and Paschoal 2019) . Also of note, we find evidence suggesting hsa-miRs -1246, -1260b, -1275, -4286, and -4454 are expressed entirely independent of canonical miRNA processing. In agreement with this possibility, hsa-miR-1246 is processed from a U2 snRNA pseudogene (Xu et al. 2019) and hsa-miR-4454 is derived from a tRNA (Reinsborough et al. 2019) and therefore actually constitutes a tsRNA ( **Figure 1F** ).

In summary, although DICER has been suggested to be involved with the expression of several tRNA, rRNA, and Y-RNA fragments (recently reviewed in (Akiyama and Ivanov 2023) ), much like a previous study carried out by our group showing that another type of miRNA-sized sncRNAs excised from snoRNAs (sdRNAs) does not require DICER (Godang et al. 2023) , the work presented here finds no evidence indicating any of the canonical miRNA biogenesis enzymes are required for the majority of tsRNA, ysRNA, and/or rsRNA expressions. That said, a number of additional tsRNAs, rsRNAs, and ysRNAs undetectable by traditional small RNAs-seq have recently been identified using new sequencing methodologies (e.g., PANDORA-seq (Shi et al. 2021) ) and it will be interesting to see if future analyses of canonical miRNA biogenesis enzyme KOs employing these methodologies find these additional sncRNAs (previously masked due to RNA modifications (Shi et al. 2021) ) behave similarly to those described in this work.

## Methods

A single FASTA file comprising all known human miRNAs contained within the miRNA registry (http://www.mirbase.org) (Kozomara and Griffiths-Jones 2014) and all known human snoRNAs, yRNAs, tRNAs, rRNAs and snRNAs currently annotated in Ensembl (Cunningham et al. 2015) was assembled. Alignments between ncRNAs and individual small RNA-seq reads were performed on the Alabama Supercomputer Center SGI UV 2000 and DMC cluster and obtained via Basic Local Alignment Search Tool (BLAST+) (Altschul et al. 1990) using the following parameters: 100% identity, word_size = 6, ungapped, and evalue = 0.001. All accepted BLAST+ alignments were restricted to perfect matches (100% identity) between 16 and 32 nts. The frequency of alignments to putative sncRNA loci across each full-length ncRNA was calculated by counting reads defined as ≥16 nts and perfect matches (100% identity) as previously defined (Alexander B. Coley et al. 2021). Publicly available next-generation small RNA deep-sequencing libraries were obtained from the NCBI Sequence Read Archive (SRA) (www.ncbi.nlm.nih.gov/sra/) (Leinonen, Sugawara, and Shumway 2011) . These included HCT116 WT (SRR22500559, SRR22500560, SRR22500569, SRR22500570, SRR22500571, SRR22500572, SRR3174960, SRR3174961, SRR3174964, SRR22319718, SRR22319724, SRR22319730), DROSHA-KO (SRR22319717, SRR22500565, SRR22500566, SRR22500567, SRR22500568, SRR22500557, SRR22500558, SRR3174962, SRR3174963, SRR22319729, SRR22319723), DGCR8-KO (+ rescue) (SRR11850241, SRR11850242, SRR11850243, SRR11850244, SRR11850245, SRR11850246), XPO5-KO (SRR3174965, SRR3174966), and DICER-KO (SRR22500555, SRR22500556, SRR3174967, SRR3174968, SRR22319734, SRR22319728, SRR22319722) small RNA transcriptomes. Individual microRNAs and sncRNAs not expressed at ≥20 RPM on average in any KO or WT library were excluded. Average BLAST-based alignment expressions for each KO/WT were confirmed by independently employing an in house algorithm, Fragment Finder (available at https://github.com/NickCampbell97/FragmentFinder-CLI ), which reported expression in reads per million (RPM) for each sncRNA and miRNA detected. RStudio was used to calculate differential expression. Returned results strictly required sncRNAs and miRNAs to be expressed at ≥ 20 RPM on average in at least one KO or WT library and all sncRNAs and miRNAs were classified as (1) upregulated; ≥ 150% expression in KO compared to WT, (2) downregulated; ≤ 50% expression in KO compared to WT, or (3) unchanged; expression in KO within 50% of WT.

## Extended Data

Description: Human miRNAs contained within the miRNA registry. Resource Type: Dataset. DOI: 10.22002/6s0gx-ypc45

Description: Human snoRNAs, yRNAs, tRNAs, and snRNAs currently annotated in Ensembl. Resource Type: Dataset. DOI: 10.22002/w11zk-n3d93
